# Prehospital Partial Resuscitative Endovascular Balloon Occlusion of the Aorta for Exsanguinating Subdiaphragmatic Hemorrhage

**DOI:** 10.1001/jamasurg.2024.2254

**Published:** 2024-07-10

**Authors:** Robbie A. Lendrum, Zane Perkins, Max Marsden, Claire Cochran, Ross Davenport, Frank Chege, Virginia Fitzpatrick-Swallow, Rob Greenhalgh, Jared M. Wohlgemut, Christine L. Henry, Ben Singer, Gareth Grier, Gareth Davies, Nick Bunker, Daniel Nevin, Mike Christian, Marion K. Campbell, Nigel Tai, Austin Johnson, Jan O. Jansen, Samy Sadek, Karim Brohi

**Affiliations:** 1 Bart’s Health National Health Service Trust, London, United Kingdom; 2 London’s Air Ambulance, London, United Kingdom; 3 Centre for Trauma Sciences, Queen Mary University of London, London, United Kingdom; 4 Academic Department of Military Surgery and Trauma, Research and Clinical Innovation, Defense Medical Services, Birmingham, United Kingdom; 5 Home Office Registered Forensic Pathologist, Wantage, United Kingdom; 6 Manx Care, Nobles Hospital, Douglas, Isle of Man; 7 Department of Critical Care Medicine, University British Columbia, Vancouver, British Columbia, Canada; 8 Health Services Research Unit, University of Aberdeen, Aberdeen, Scotland; 9 University of Utah Health, Salt Lake City; 10 Department of Surgery, University of Alabama at Birmingham

## Abstract

**Importance:**

Hemorrhage is the most common cause of preventable death after injury. Most deaths occur early, in the prehospital phase of care.

**Objective:**

To establish whether prehospital zone 1 (supraceliac) partial resuscitative endovascular balloon occlusion of the aorta (Z1 P-REBOA) can be achieved in the resuscitation of adult trauma patients at risk of cardiac arrest and death due to exsanguination.

**Design, Setting, and Participants:**

This was a prospective observational cohort study (Idea, Development, Exploration, Assessment and Long-term follow-up [IDEAL] 2A design) with recruitment from June 2020 to March 2022 and follow-up until discharge from hospital, death, or 90 days evaluating a physician-led and physician-delivered, urban prehospital trauma service in the Greater London area. Trauma patients aged 16 years and older with suspected exsanguinating subdiaphragmatic hemorrhage, recent or imminent hypovolemic traumatic cardiac arrest (TCA) were included. Those with unsurvivable injuries or who were pregnant were excluded. Of 2960 individuals attended by the service during the study period, 16 were included in the study.

**Exposures:**

ZI REBOA or P-REBOA.

**Main Outcomes and Measures:**

The main outcome was the proportion of patients in whom Z1 REBOA and Z1 P-REBOA were achieved. Clinical end points included systolic blood pressure (SBP) response to Z1 REBOA, mortality rate (1 hour, 3 hours, 24 hours, or 30 days postinjury), and survival to hospital discharge.

**Results:**

Femoral arterial access for Z1 REBOA was attempted in 16 patients (median [range] age, 30 [17-76] years; 14 [81%] male; median [IQR] Injury Severity Score, 50 [39-57]). In 2 patients with successful arterial access, REBOA was not attempted due to improvement in clinical condition. In the other 14 patients (8 [57%] of whom were in traumatic cardiac arrest [TCA]), 11 successfully underwent cannulation and had aortic balloons inflated in Z1. The 3 individuals in whom cannulation was unsuccessful were in TCA (failure rate = 3/14 [21%]). Median (IQR) pre-REBOA SBP in the 11 individuals for whom cannulation was successful (5 [46%] in TCA) was 47 (33-52) mm Hg. Z1 REBOA plus P-REBOA was associated with a significant improvement in BP (median [IQR] SBP at emergency department arrival, 101 [77-107] mm Hg; 0 of 10 patients were in TCA at arrival). The median group-level improvement in SBP from the pre-REBOA value was 52 (95% CI, 42-77) mm Hg (*P* < .004). P-REBOA was feasible in 8 individuals (8/11 [73%]) and occurred spontaneously in 4 of these. The 1- and 3-hour postinjury mortality rate was 9% (1/11), 24-hour mortality was 27% (3/11), and 30-day mortality was 82% (9/11). Survival to hospital discharge was 18% (2/11). Both survivors underwent early Z1 P-REBOA.

**Conclusions and Relevance:**

In this study, prehospital Z1 P-REBOA is feasible and may enable early survival, but with a significant incidence of late death.

**Trial Registration:**

ClinicalTrials.gov Identifier: NCT04145271

## Key Points

**Question:**

Is it feasible to deploy prehospital zone 1 (supraceliac) partial resuscitative endovascular balloon occlusion of the aorta (Z1 P-REBOA) in the prehospital resuscitation of adult trauma patients at risk of cardiac arrest and death due to exsanguination?

**Findings:**

In this cohort study of 16 patients with severe injuries and shock, prehospital Z1 P-REBOA was delivered successfully in 8 of the 11 patients who underwent Z1 REBOA. This strategy was associated with improved proximal blood pressure and early mortality.

**Meaning:**

The findings indicate that in the prehospital resuscitation of adult trauma patients at risk of cardiac arrest and death due to exsanguination, Z1 P-REBOA is feasible and may enable early survival, but with a significant incidence of late death.

## Teaser

This cohort study evaluates the use of prehospital partial resuscitative endovascular balloon occlusion of the aorta in injured patients at risk of exsanguination.

## Introduction

Uncontrolled bleeding is the most common cause of preventable death after injury and is responsible for approximately one-third of all trauma-related mortality.^1,2,3,4,5^ Most of these deaths, both in civilian and military settings, occur rapidly from exsanguination—typically within 30 minutes of injury and well before most patients can reach the hospital.^6,7,8^ For those that do reach the hospital, mortality is highest in the first 3 to 72 hours, attributable to the consequences of early and profound hemorrhagic shock.^8,9^ Most deaths are due to noncompressible torso hemorrhage, and yet there are no established solutions for temporizing this bleeding in the prehospital or far-forward environment.

Resuscitative endovascular balloon occlusion of the aorta (REBOA) is a technique for the temporary proximal control of subdiaphragmatic torso hemorrhage.^10^ Balloon occlusion of the descending thoracic aorta (zone 1 [Z1], supraceliac) reduces distal blood loss while also improving coronary and cerebral perfusion.^11,12^ This intervention can temporarily delay cardiac arrest from exsanguination, potentially extending the time window to provide definitive treatment.^13^ However, in the UK, the average time taken to reach a hospital following major injury is 90 minutes, long after the window of potential benefit.^3,14^

In the prehospital setting, where there is potentially the most benefit from earlier, temporary truncal hemorrhage control, we have previously shown that it is possible to deploy REBOA.^15,16^ However, this was limited to infrarenal placement (Z3) to avoid prolonged visceral ischemia.^15^ To further investigate this resuscitation strategy in this setting, it is important to establish whether it can be delivered in patients with more proximal hemorrhage in the abdomen (Z1 REBOA) and to achieve maximal increase in proximal blood pressure (BP) and therefore myocardial perfusion in patients with profound shock and traumatic cardiac arrest (TCA) caused by subdiaphragmatic exsanguinating hemorrhage.^10,13,17^ However, this comes with an increased risk of visceral ischemic injury.

Partial REBOA (P-REBOA) describes allowing titrated low-volume aortic flow past the balloon. It can provide a balance by creating a region of distal permissive hypotension, thus reducing hemorrhage, while mitigating distal ischemia or reperfusion injury and simultaneously augmenting proximal hemodynamics.^18,19^ To our knowledge, Z1 P-REBOA has not previously been deployed in the prehospital environment.

The overall aim of this study was to explore the potential use of prehospital Z1 P-REBOA in patients with immediately life-threatening noncompressible torso hemorrhage. Our first objective was to determine whether it was feasible to deploy prehospital Z1 REBOA in critically bleeding trauma patients. The second objective was to determine the feasibility of delivering controlled P-REBOA in these patients and assess technical performance. Finally, we wished to explore the potential impact of Z1 P-REBOA on prehospital and in-hospital clinical outcomes and safety.

## Methods

### Study Design

We performed a prospective observational cohort study of prehospital Z1 P-REBOA delivered by a physician-led prehospital team in an advanced urban trauma system including injured patients with immediately life-threatening subdiaphragmatic hemorrhage. The study is a sequentially reported case series, adhering to the Idea, Development, Exploration, Assessment and Long-term follow-up (IDEAL) 2A framework for surgical innovation.^20^ Patients were followed up until discharge, death, or for 90 days from the date of the final patient’s admission to hospital. Due to the emergency context, enrollment proceeded without consent. Consent for continued participation was sought once patients were no longer in a life-threatening condition. Ethics approval was granted by the London–Southeast Research Ethics Committee and the Health Research Authority and sponsored by Barts Heath National Health Service Trust. All cases were reviewed by a multidisciplinary investigative committee, which provided oversight and guidance for the study management group.

### Setting

London’s Air Ambulance provides a 24-hour dedicated advanced trauma service to Greater London, working in conjunction with the London Ambulance Service. The physician-paramedic team attends approximately 2000 injured patients per year and can deliver advanced prehospital interventions, including prehospital anesthesia, blood transfusion, resuscitative thoracotomy, and REBOA (Z3, since 2014).^15,21^ Severely injured patients are transported to 1 of London’s 4 major trauma centers.

### Inclusion and Exclusion Criteria

Eligible participants for enrollment were trauma patients aged 16 years and older and attended by London’s Air Ambulance with a clinical diagnosis of exsanguinating subdiaphragmatic hemorrhage, with recent or imminent risk of hypovolemic cardiac arrest thought to be amenable to treatment with Z1 REBOA. Exclusion criteria were patients suspected to be younger than 16 years, unsurvivable injuries, or pregnancy. Patients entered the study at the point an attempt was made at common femoral artery access for Z1 REBOA.

### Prehospital REBOA

Z1 REBOA was performed as an adjunct to standard trauma care via an 8F access sheath (Avanti; Cordis) inserted percutaneously into the common femoral artery under ultrasound guidance and using the ER-REBOA catheter (Prytime Medical Devices). Arterial BP was measured distal to the balloon (common femoral artery, sidearm access sheath) and proximal to the balloon (thoracic aorta, catheter tip). Catheter insertion depth was determined by measuring external landmarks (cannulation site to mid sternum) targeting approximately 45 cm.^22,23^ The balloon was inflated using 0.9% saline until the distal pulsatile pressure trace (measured from the access sheath sidearm) became nonpulsatile and proximal pressure improved.^19^

Pressure measured distal to the site of balloon occlusion is directly proportional to flow, exhibiting a linear relationship.^18^ An increase in the distal mean arterial pressure of 5 to 10 mm Hg above the postinflation baseline is associated with an increase in distal flow of 250 to 500 mL/min.^18,19^ Therefore, when instituting P-REBOA, distal pressure can be targeted as a surrogate for flow.

### Outcomes

Successful prehospital Z1 REBOA was defined as the composite end point of balloon insertion to 35 to 55 cm, proximal arterial BP transduced, and balloon inflation. Successful prehospital P-REBOA was defined as the composite end point of evidence of an increase in distal mean arterial pressure of at least 5 to 10 mm Hg above the postinflation baseline and/or a return of distal pulsatility, or distal pulsatility never absent postnitial balloon inflation.

Clinical end points included systolic BP (SPB) response to Z1 REBOA, incidence of prehospital traumatic cardiac arrest, mortality rate (1 hour, 3 hours, 24 hours, or 30 days postinjury), cause of death, and survival to hospital discharge. All adverse events related to patient injury, resulting critical illness, and treatment as well as femoral cannulation, REBOA catheter insertion, and/or the anticipated effects of aortic occlusion were predefined and recorded.

### Data Collection

Temporal, physiological, and clinical data were prospectively collected and recorded using REDCap.^24^ Physiological data were downloaded directly from the patient monitor (ZOLL X-series; Zoll Medical). Other data were obtained from original sources, including standard prehospital and in-hospital patient records, blood tests, imaging, and postmortem data. Injuries were classified according to the Abbreviated Injury Scale 2005 and Injury Severity Score by certified coders.^25,26^

### Statistical Analysis

Continuous data are reported as medians with IQRs and categorical data as frequency and percentage. Paired data were compared using the Wilcoxon matched-pairs signed rank test (PRISM version 10; GraphPad). Statistical significance was set as a 2-tailed *P* value less than .05.

## Results

During the 21-month study period (June 2020 through to March 2022), of 2960 individuals attended by the service, arterial access for Z1 REBOA was attempted in 16 patients (median [range] age, 30 [17-76] years; 13 [81%] male and 3 [19%] female). Most injuries (13/16 [81%]) were caused by high-energy blunt trauma (Table 1). The median (IQR) time from injury to London’s Air Ambulance arrival on scene was 21 (16-28) minutes. Patients were critically injured, with a median (IQR) Injury Severity Score of 50 (39-57), and were profoundly hypotensive, with a median (IQR) initial SBP measured by London’s Air Ambulance of 58 (47-82) mm Hg and diastolic BP (DBP) of 35 (27-49) (Table 1). One patient had unrecordable noninvasive BP but a palpable central pulse. Three patients were in TCA (Tables 1 and 2).

**Table 1.** Baseline and Outcome Characteristics

Demographic characteristics	Other characteristics					Timing/trajectory			Outcome	
Patient No. (sequential order)	Mechanism of injury	ISS	First BP measurement (mean arterial pressure), mm Hg	First HR measurement, bpm^a^	Initial GCS score	Injury to LAA arrival, min	TCA preintervention	Z1 P-REBOA		Discharge from hospital alive
**Patients who underwent Z1 REBOA**										
1 (1)	Stabbing proximal thigh/flank	32	57/12 (20)	39^b^	3	44	Yes	Yes		Yes
2 (2)	Fall, 13-m bridge	50	59/39 (48)	170	9	18	Yes^c^	No		No
3 (3)	Fall, 22-m building	57	58/28 (40)	141	14	12	No	Yes		No
4 (4)	Motorcyclist collision with bus stop	55	47/29 (35)	131	9	25	No	Yes		No
5 (9)	Motorcyclist collision with lamppost	57	85/39 (56)	78	14	19	No	Yes		No
6 (10)	Crushed by 2 cars	16	103/76 (85)	136	9	29	No	Yes		No
7 (11)	Pedestrian hit by train	66	47/31 (38)	153	3	43	Yes^d^	Yes		No
8 (12)	Fall, 18-m building	PLE	TCA	33^b^	3	24	Yes	No		No
9 (14)	Fall, 22-m building	50	26/17 (20)	156	11	17	No	Yes		No
10 (15)	Motorcyclist collision with lamppost	50	74/50 (60)	120	15	21	Yes	No		No
11 (16)	Fall, 48-m building	50	U/R NIBP	161	4	13	No	Yes		Yes
**Failed attempt CFA access; Z1 REBOA attempt**										
12 (5)	Buttock stabbing	16	TCA	155^b^	3	16	Yes	No		No
13 (6)	Fall, 18-m building	43	32/27 (29)	69^b^	3	16	Yes	No		No
14 (13)	Groin stabbing	PLE	TCA	10^b^	3	45	Yes	No		No
**CFA access only; no Z1 REBOA attempt due to improvement in clinical condition, patient no longer met inclusion criteria**										
15 (7)	Pedestrian hit by train	59	58/46 (52)	51	7	23	No	No		Yes
16 (8)	Fall, 30-m building	41	99/63 (75)	80	14	21	No	No		Yes

Abbreviations: BP, blood pressure; GCS, Glasgow Coma Scale; HR, heart rate; ISS, Injury Severity Score; LAA, London’s Air Ambulance; N/R, no REBOA—not attempted due to clinical improvement or not recorded; NIBP, noninvasive BP; PEA, pulseless electrical activity; PLE, pronounced life extinct; P-REBOA, partial REBOA; REBOA, resuscitative endovascular balloon occlusion of the aorta; TCA, traumatic cardiac arrest; U/R, unrecordable; Z, zone.

^a^ Includes PEA.

^b^ Pulseless electrical activity.

^c^ Described immediately postfall from height, responded to initial cardiopulmonary resuscitation, then rearrested pre-REBOA.

^d^ Loss of central pulse immediately pre-REBOA.

**Table 2.** Clinical Outcomes

Patient No. (sequential order)	Proximal hemodynamic response (mean arterial pressure)			Cardiac arrest		MODS, SOFA score at admission	Length of stay, d		Survival				CPC
	Pre-REBOA BP	Post-REBOA BP	Arrival at ED BP	Prehospital TCA	RT		Critical care	Hospital	3 h	24 h	30 d	90 d or Discharge	
**Patients who underwent Z1 REBOA**													
1 (1)	53/10 (21)	96/29 (51)	105/85 (94)	Yes	No	13	38	62	Yes	Yes	Yes	Yes	1
2 (2)	24/16 (20)	TCA, M^a^	66/41 (49)	Yes^b^^,^^c^	Yes^d^^,^^e^	13	<1	<1	Yes	No	No	No	NA
3 (3)	52/37 (30)	105/82 (89)	106/57 (90)	No	No	12	2	2	Yes	Yes	No	No	NA
4 (4)	42/13 (27)	79/53 (62)	110/66 (78)	No	No	4	12	12	Yes	Yes	No	No	NA
5 (9)	25/15 (19)	54/28 (37)	102/73 (79)	No	No	17	2	2	Yes	Yes	No	No	NA
6 (10)	40/28 (32)	67/48 (58)	131/71 (94)	No	No	12	2	2	Yes	Yes	No	No	NA
7 (11)	47/31 (38)	111/71 (84)^f^	99/46 (65)^f^	Yes^b^	No	13	11	11	Yes	Yes	No	No	NA
8 (12)	TCA, M^g^	114/38 (38)^h^	PLE	Yes^b^^,^^c^	No	PLE	0^i^	0	No	No	No	No	NA
9 (14)	51/29 (34)	56/46 (52)	80/67 (75)	No	No	13	2	2	Yes	Yes	No	No	NA
10 (15)	TCA, M^a^	74/54 (64)	69/52 (58)	Yes^b^^,^^c^	Yes^d^^,^^j^	13	<1	<1	Yes	No	No	No	NA
11 (16)	48/39 (43)	104/78 (88)	100/81 (90)	No	No	12	39	76	Yes	Yes	Yes	Yes	1
**Failed CFA access**													
12 (5)	F/A	F/A	99/60 (73)	Yes	Yes^e^^,^^k^	17	<1	<1	Yes	No	No	No	NA
13 (6)	F/A	F/A	52/27 (37)	Yes	Yes^e^^,^^k^	15	0^l^	0	Yes	No	No	No	NA
14 (13)	F/A	F/A	PLE	Yes	Yes^e^^,^^k^	PLE	0^m^	0	No	No	No	No	NA
**CFA access only**													
15 (7)	N/R	N/R	114/71 (81)	No	No	13	46	246	Yes	Yes	Yes	Yes	1
16 (8)	N/R	N/R	143/69 (82)	No	No	4	14	29	Yes	Yes	Yes	Yes	1

Abbreviations: BP, blood pressure; CPC, cerebral performance category score at hospital discharge; ED, emergency department; F/A, failed arterial access, M, missing data; MODS, multiple organ dysfunction syndrome; NA, not applicable; N/R, no REBOA—not attempted due to improvement; PHRT, resuscitative thoracotomy; PLE, pronounced life extinct; REBOA, resuscitative endovascular balloon occlusion of the aorta; RT, resuscitative thoracotomy; SOFA, sequential organ failure assessment; TCA, traumatic cardiac arrest; Z, zone.

^a^ Proximal arterial BP transduced post-REBOA, monitoring setup issue (failed transducer zero), patient in TCA.

^b^ TCA occurred pre-REBOA.

^c^ TCA persisted or recurred transiently post-REBOA.

^d^ RT indication: predicted intrathoracic pathology.

^e^ Location of RT intervention: prehospital.

^f^ Noninvasive BP value, proximal transducer failure.

^g^ Monitoring issue, proximal intra-aortic balloon pump transduced post-REBOA (transducer cable disconnection), patient in TCA.

^h^ External cardiac massage, established TCA.

^i^ Died prehospital.

^j^ Location of RT intervention: operating theater.

^k^ RT indication: failed femoral arterial access, exsanguination, and aortic control.

^l^ Died in operating theater.

^m^ Died in ED.

### Feasibility

Of the 16 patients, the clinical condition of 2 improved and REBOA was not attempted. In the other 14 patients who had an attempt at Z1 REBOA, all demonstrated ongoing hemodynamic deterioration despite blood product transfusion, with 8 patients in TCA by the time of intervention (57%). Three patients (all in TCA) had failed attempts at femoral arterial access (21%) due to an inability to visualize the common femoral artery with ultrasonography. All 3 underwent immediate resuscitative thoracotomy for open aortic control. The 11 remaining patients (5 of 11 in TCA [46%]) had REBOA catheters inserted in Z1. There were initial, technical arterial BP-monitoring issues in 2 of these patients (both in TCA from the point of clinical contact), leaving 9 patients with a measured pre-REBOA median (IQR) SBP of 47 (33-52) mm Hg and DBP of 28 (14-34) mm Hg.

### Z1 Balloon Occlusion

**Figure 1.**
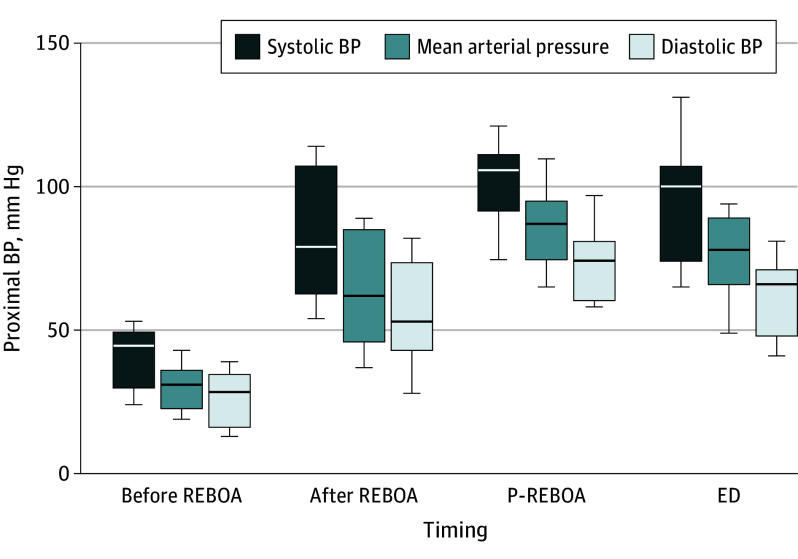
Group-Level Hemodynamic Response to Zone 1 (Z1)–Resuscitative Endovascular Balloon Occlusion of the Aorta (REBOA) and Partial (P)–REBOA The horizontal lines indicate medians; boxes indicate IQRs, and whiskers show maximum values. BP indicates blood pressure; ED, emergency department.

**Figure 2.**
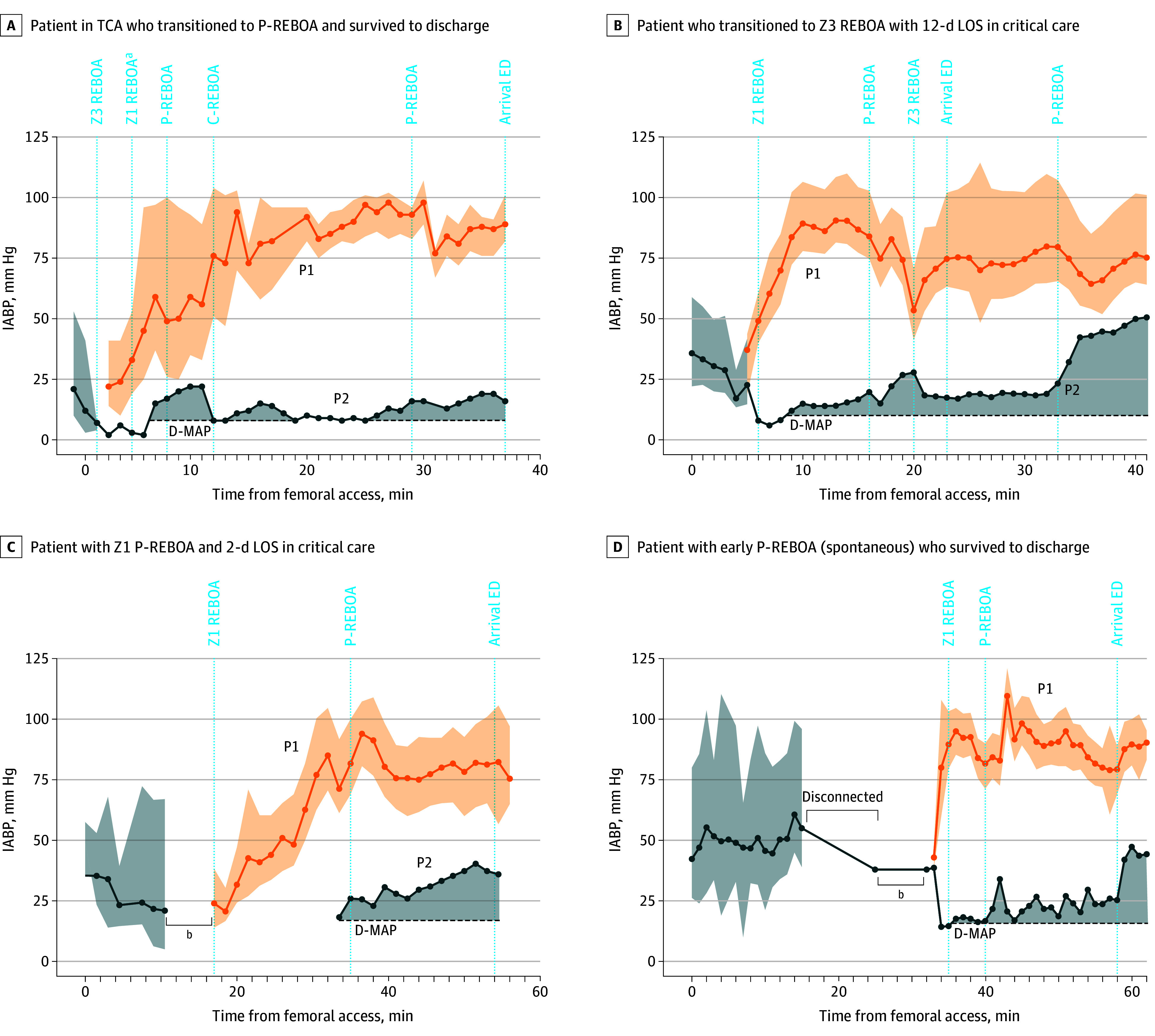
Individual Hemodynamic Responses to Zone 1 (Z1)–Resuscitative Endovascular Balloon Occlusion of the Aorta (REBOA) and Partial (P)-REBOA C-REBOA indicates complete REBOA; LOS, length of stay; D-MAP, postinflation distal mean arterial pressure baseline; P1, proximal (aortic) mean arterial blood pressure and pulse pressure measured from the tip of the REBOA catheter; P2, distal (common femoral artery) mean arterial pressure measured from the side arm of the 8F access sheath; TCA, traumatic cardiac arrest. ^a^External cardiac massage epinephrine, 100 μg. ^b^Sheath upsize 4F (early femoral access for arterial blood pressure measurement) to 8F (REBOA arterial access sheath).

All catheters were inserted to 45 cm except 1 (47 cm). Proximal arterial (aortic) pressure was transduced in 10 of the 11 patients undergoing REBOA at the point of balloon inflation and all balloons were inflated. Therefore, the proportion of patients in whom Z1 REBOA was delivered as per definition was 91% (10 of 11). The median (IQR) time from patient injury (emergency call) to balloon inflation in Z1 was 57 (54-67) minutes. All procedures were performed at the scene of the injury except 1, which was performed during ambulance transfer to the hospital. Balloon occlusion was associated with significant improvement in BP; the 10 patients in whom BP was successfully measured, the median (IQR) post-REBOA SBP was 88 (64-107) mm HG and DBP was 51 (36-73) mm Hg (Figure 1). Median (IQR) group-level improvements in SBP and DBP were 40 mm Hg (95% CI, 5-64; *P* = .008) and 30 mm Hg (95% CI, 13-45; *P* = .008), respectively (Figures 1 and 2; Table 2; eFigure 1 and eTable 1 in Supplement 1).

### P-REBOA

P-REBOA was achieved in 8 of the 11 patients who underwent Z1 REBOA (73%). In 7 of these individuals, P-REBOA was instituted after a period of complete REBOA (C-REBOA) for a median (IQR) duration of 8 (3-18) minutes. The median (IQR) distal mean arterial pressure after institution of P-REBOA was 22 (20-25) mm Hg, from the immediate post–balloon inflation distal median (IQR) arterial pressure of 13 (7-15) mm Hg (median of differences, 10 mm Hg; 95% CI, 5-30; *P* = .02). In 1 patient, P-REBOA was used throughout the period of intervention. P-REBOA occurred spontaneously without the requirement for balloon deflation in 4 of the 8 patients undergoing P-REBOA. In the others, the median (IQR) volume removed to initiate P-REBOA was 1.0 (0.6-1.8) mL. In all 8 individuals who underwent P-REBOA, distal pulsatility on the arterial waveform was observed with the increase in distal mean arterial pressure (Table 1; Figures 1 and 2; eTable 1 and eFigure 1 in Supplement 1).

Proximal hemodynamic stability was maintained in 7 of the 8 patients during institution and maintenance of P-REBOA. The 3 patients who underwent Z1 REBOA in whom P-REBOA was not possible had associated severe ongoing proximal hypotension and therefore an inability to generate partial flow either spontaneously or by balloon deflation (Figures 1 and 2; Tables 1 and 2; eFigure 1 and eTable 1 in Supplement 1).

### Prehospital Course

The median (IQR) time from injury (emergency call) to emergency department arrival was 89 (82-108) minutes. Continued improvement in proximal BP was observed compared with the pre-REBOA values, with a median (IQR) emergency department arrival SBP (in the 10 patients transported to the hospital) of 101 (77-107) mm Hg and DBP of 67 (51-75) mm Hg. Median group level improvements in SBP and DBP from preinflation values were 52 mm Hg (95% CI, 42-77; *P* = .004) and 42 mm Hg (95% CI, 20-58; *P* = .004), respectively. Three patients remained hypotensive (SBP <90 mm Hg), and 1 patient died on scene and therefore was not transported. External cardiac massage was performed post–Z1 REBOA in 3 individuals due to established TCA. This led to a sustained return of spontaneous circulation in 1 individual, with the patient surviving to hospital discharge (Figure 2). Four transient adverse events related to pressure transducer setup and operation and 1 to balloon deflation (5 of 11 cases [46%]) were reported (Figures 1 and 2; Table 2; eTables 1 and 2 in Supplement 1).

### In-Hospital Management and Outcome

Definitive hemorrhage control was achieved with immediate damage-control surgery or angioembolization in 9 of the 11 patients undergoing Z1 REBOA. Of the remaining 2 patients, 1 underwent emergent orthopedic surgery and neurosurgery and 1 died on scene and therefore did not access in-hospital treatment. Ten of the 11 patients undergoing Z1 REBOA survived for more than 3 hours postinjury (1- and 3-hour mortality rate, 9%). Eight patients survived for 24 hours (24-hour mortality rate, 27%), and 2 patients survived for 30 days and to hospital discharge (30-day mortality rate, 82%; survival to hospital discharge, 18%). Both survivors underwent early Z1 P-REBOA and therefore survival to hospital discharge in the 8-patient Z1 P-REBOA cohort was 25% (2/8) (Tables 2 and 3; eFigure 2 in Supplement 1).

**Table 3.** In-Hospital Management, Cause, and Pathophysiological Mode of Death and Safety

Patient No. (sequential order)	Index hemorrhage-control intervention	Other procedure	Cause of death^a^^,^^b^	Pathophysiological mode of death^b^	Safety, morbidity, and complications
**Patients who underwent Z1 REBOA**					
1 (1)	DCS laparotomy, packing^c^	Repeat laparotomy (multiple)	NA	NA	AKI, CRRT acute intestinal ischemia, bowel resection,^d^^,^^e^ MODS
2 (2)	Laparotomy, hepatic packing	Nephrectomy, hemicolectomy	1a, Hemorrhage, hypovolemic shock; 1b, portal vein laceration/grade 4 liver laceration, renal artery injury	MODS, CS, EH	Acute intestinal ischemia,^d^ bowel resection
3 (3)	Laparotomy extraperitoneal pelvic packing; angioembolization IIAs bilaterally and lumbar artery	Repeat laparotomy/extraperitoneal pelvic packing, nasal packing epistaxis, ICP bolt insertion, debridement/stabilization bilateral open hindfoot fractures	1a, Hemorrhage, hypovolemic shock; 1b, vertical shear pelvic fracture, bilateral internal iliac and left lumbar artery lacerations	MODS, CS, EH	AKI, CRRT, EIA cannulation, sheath removal, surgical closure arteriotomy
4 (4)	Femoral traction pins, bilateral SI screws and anterior external fixation, laparotomy, surgical control gluteal arterial injury	Open forearm fracture ORIF; percutaneous lumbar spinal fracture fixation	1a, ARDS, hypoxic ischemic encephalopathy; 1b, pneumonia, hemorrhage; 1c, comminuted unstable open pelvic fracture, kidney laceration/avulsion; 2, pulmonary contusions	Hypoxia, cerebral/edema, cerebellar tonsillar herniation	AKI, CRRT, MOD, right atrial thrombus
5 (9)	Laparotomy and 4-quadrant packing	Repeat laparotomy (×2) and clamshell thoracotomy	1a, Uncontrolled hemorrhage; 1b, grade 5 liver laceration	MODS, CS, EH	AKI, CRRT, distal arterial thrombus/embolism requiring intervention (CFA thrombectomy or embolectomy)
6 (10)	DCS lower limbs bilaterally, completion traumatic AKIs	None	1a, Hemorrhage (bilateral high SFA transections); 1b, severe bilateral crush injuries lower limbs, traumatic AKIs	MODS, vasoplegic shock	AKI, CRRT, liver dysfunction/failure, bilateral AKAs
7 (11)	Laparotomy with CIA clamp	DCS lower limbs bilaterally, completion traumatic AKAs; ICP bolt: craniectomy, evacuation hematoma^f^	1a, Hypoxic ischemic encephalopathy; 1b, TBI, hemorrhage-associated high bilateral traumatic lower limb amputations	TBI, exsanguination	Bilateral AKAs, MODS
8 (12)	PLE	PLE	1a, Hemorrhage, complex pelvic fracture, TBI	CS, EH, TBI	NA
9 (14)	Open right distal femur, tibia fracture: external fixator/wound debridement; open right humerus fracture: external fixator/wound debridement	Decompressive craniectomy evacuation of SDH and ICP bolt	1a, TBI and craniofacial disruption, hemorrhage—complex pelvic fracture, bilateral open femoral fractures	TBI, MODS, CS	AKI
10 (15)	Thoracotomy	Nil	1a, Hemorrhage; 1b, right renal artery laceration	MODS, CS, EH	Nil
11 (16)	Laparotomy extra peritoneal pelvic packing	Angioembolization IIA and femoral arterial embolectomy; repeat laparotomy—bilateral IIA ligation	NA	NA	AKI, CRRT, MODS, distal arterial thrombus formation/embolism-thrombectomy, embolectomy
**Failed attempt CFA access; Z1 REBOA attempt**					
12 (5)	Angioembolization IIA	Closure clamshell thoracotomy	1a, Hemorrhage; 1b, stab wound left IIA	MODS, CS, EH	AKI, acute intestinal ischemia,^d^ CFA dissection
13 (6)	Laparotomy and 4-quadrant packing, hepatic packing, extraperitoneal pelvic packing	In-hospital Z1 REBOA; VA ECMO cannulation	1a, Hemorrhage; 1b, severe liver laceration, vertical shear fracture pelvis	MODS, CS, EH	AKI, CRRT
14 (13)	PLE	PLE	1a, Hemorrhage; 1b, stab wound right iliac vein	MODS, CS, EH	Nil
**CFA access only; no Z1 REBOA attempt due to improvement in clinical condition**					
15 (7)	Angioembolization IIA bilaterally	ORIF comminuted pelvic fracture, ICP bolt; MUA nasal bones, ORIF mandible; multiple ORIF, LUL, LLL; surgical tracheostomy	NA	NA	Nil
16 (8)	None	ORIF humerus and calcaneus	NA	NA	Nil

Abbreviations: AKA, above-knee amputation; AKI, acute kidney injury; ARDS, acute respiratory distress syndrome; CFA, common femoral artery; CIA, common iliac artery; CRRT, continuous renal replacement therapy; CS, cardiogenic shock; DCS, damage-control surgery; EH, exsanguinating hemorrhage; EIA, external iliac artery; ICP, intracranial pressure; IIA, internal iliac artery; LLL, left lower lobe; LUL, left upper lobe; MODS, multiorgan dysfunction syndrome; MUA, manipulation under anesthetic; NA, not applicable; ORIF, open reduction internal fixation; PLE, pronounced life extinct; REBOA, resuscitative endovascular occlusion of the aorta; SFA, superficial femoral artery; SI, sacroiliac; TBI, traumatic brain injury; VA ECMO, venoarterial extracorporeal membrane oxygenation.

^a^ Cause of death classification: 1a indicates the disease or condition immediately causing death; 1b, the underlying cause of 1a; 1c, the underlying cause of 1b; 2, any disease or condition that did not cause death but contributed in some way.

^b^ Cause of death and pathophysiological mode of death agreed by a multidisciplinary consensus process (forensic pathologist, trauma surgeon, intensive care medicine specialist, anesthesiologist, emergency medicine physician, prehospital care physician).

^c^ Packing, not further specified.

^d^ Acute intestinal ischemia defined as evidence of gut necrosis at laparotomy or findings on computed tomography consistent with gut ischemia in the context of elevated blood lactate.

^e^ Iatrogenic bowel injury during initial procedure due to adhesions (previous laparotomy).

^f^ Iatrogenic injury ICP bolt insertion leading to acute intracranial hematoma and raised ICP.

### Safety

Multiple organ dysfunction syndrome (≥2 organ system failures) was evident in 10 individuals (10/11 [91%]). Acute kidney injury (Kidney Disease: Improving Global Outcomes Criteria Stage 3) was present in 7 individuals (7/11 [64%]), with 6 (6/11 [55%]) requiring continuous renal replacement therapy. Both survivors remained free of continuous renal replacement therapy at hospital discharge. Acute intestinal ischemia was observed in 2 patients (2/11 [18%]) with both patients requiring bowel resection. Distal arterial thrombus formation requiring intervention (thrombectomy) was observed in 2 patients (2/11 [18%]), and 2 different patients received bilateral above-knee amputations (2/11, [8%]). In both cases, these procedures were completion traumatic above-knee amputations (Table 3).

## Discussion

This cohort study reports the initial experience of ZI P-REBOA as a prehospital resuscitation strategy for patients with exsanguinating subdiaphragmatic hemorrhage following injury. Prehospital Z1 REBOA was feasible and associated with significant improvements in proximal BP. Z1 P-REBOA as a means of mitigating distal ischemia was implemented successfully in most cases and tolerated with proximal hemodynamic stability. The cohort of patients who met the inclusion criteria were more severely injured (median Injury Severity Score, 50) than in our previously reported observational study^15^ of prehospital Z3 REBOA (median Injury Severity Score, 34) and other in-hospital studies of REBOA.^27,28^

All patients displayed a positive hemodynamic response to Z1 REBOA. This response was gradual, with 45% remaining hypotensive (SBP <90 mm Hg) in the immediate post-REBOA phase with general recovery in proximal BP in most by emergency department arrival, and is in contrast to the often immediate BP response seen with REBOA in animal models of hypovolemic shock.^17,18^ This may reflect profound myocardial hypoperfusion in the immediate postinjury phase and the development of concurrent cardiogenic shock. This cohort had DBP levels below that required to allow effective coronary perfusion (median DBP preintervention, 28 mm Hg) and were therefore at high risk of myocardial ischemia, asystole, and TCA.^29^ Z1 REBOA was associated with an immediate increase in DBP to levels adequate to restore coronary perfusion.^30^ There were no instances of proximal hypertension in response to Z1 REBOA, which have been reported in large animal models to lead to hyperemic coronary blood flow, high left ventricular wall tension, and myocardial injury.^12^

P-REBOA was feasible in all patients who exhibited improved proximal BP post-REBOA, and the transition from C-REBOA to P-REBOA happened in less than 10 minutes. P-REBOA was observed to occur spontaneously in half of the patients. Several factors are likely to be important. First, procedurally, the balloon was inflated until the distal pulsatile trace disappeared, avoiding overinflation. Second, increased proximal BP (cardiac recovery, preload restoration, improved stroke volume and cardiac output) may have contributed. Increased aortic capacitance or diameter may have also been a factor. In individuals in whom a reduction of balloon volume was required to initiate P-REBOA, the volume removed was relatively small and again reflects the procedure (avoiding balloon overinflation). P-REBOA was not feasible in 3 individuals due to severe ongoing proximal hypotension, with 1 remaining in TCA postintervention. The prolonged period of complete aortic occlusion in 2 patients occurred due to an inability to generate a proximal pressure sufficient to deliver or establish P-REBOA or enable balloon deflation.

Three-fourths of patients survived for 24 hours postinjury despite more than half experiencing prehospital TCA due to exsanguination. However, survival to 30 days was low (2/11 [18%]), with most patients dying with clinical evidence of multiple organ dysfunction syndrome and cardiogenic shock. Early extracorporeal membrane oxygenation for patients with major injury and cardiogenic shock is now increasingly available.^31,32^ It is possible that earlier diagnosis of cardiogenic shock and early reperfusion with early extracorporeal membrane oxygenation could have led to survival in some cases.

Both survivors underwent early and sustained P-REBOA (occurring spontaneously in 1 patient), and both had total REBOA times significantly longer than 60 minutes (majority P-REBOA). This is in contrast to previous reports suggesting that there are no survivors beyond 60 minutes of C-REBOA and is likely to reflect the impact of P-REBOA mitigating distal ischemia, albeit this is dependent on the recovery of proximal BP.^33,34,35^

The UK-REBOA trial represents the first in-hospital randomized clinical trial of REBOA. It reported that the addition of a REBOA strategy to standard care, incurring a 20-minute delay to definitively control hemorrhage, increased 90-day mortality with high probability.^36^ This finding is important: the time taken to perform REBOA at the point of injury is likely to increase prehospital time and therefore the time to definitively control hemorrhage, with potential negative impacts on survival.^37^ Conversely, it is possible that a significant proportion of the patients in the present study may have died prior to accessing definitive care in hospital without advanced intervention and will continue to do so without efforts to temporize exsanguinating hemorrhage in the prehospital phase of care.^6,7,8,9,14,38^ Further investigation into identifying specific pathophysiological signatures indicative of early death, to potentially improve patient selection, and to allow earlier targeted intervention in those most likely to benefit while avoiding delay in those who may not, is warranted.

### Strengths and Limitations

Although the study has a small sample size, it represents most cases of this type performed worldwide. Arterial BP monitoring setup errors occurred in several cases, therefore limiting the number of complete datasets for analysis. Data pertaining to blood product transfusion volume were not collected. This was an early-stage, single-arm observational study of prehospital Z1 P-REBOA focused on technical, short-term clinical and safety-related outcomes only and therefore is not designed to evaluate the effectiveness of this strategy in comparison with standard, physician-delivered prehospital care.

## Conclusions

In this study, prehospital Z1 P-REBOA using distal pressure as a surrogate for flow was feasible for the resuscitation of exsanguinating trauma patients at risk of imminent prehospital death. This strategy was associated with increased proximal BP, was tolerated with ongoing proximal hemodynamic stability, and may enable early survival, but with a significant incidence of multiple organ dysfunction syndrome and late death.

## Notes

Supplement 1. eFigure 1. Individual Hemodynamic responses to Z1-REBOA and P-REBOA eFigure 2. Kaplan-Meier Survival Estimates Z1 REBOA, P-REBOA and Resuscitative Thoracotomy eTable 1. Technical Outcomes – Z1 REBOA Patients eTable 2. Key Timings (minutes)

Supplement 2. Data sharing statement
