# Supplementary material for: Prehospital Partial Resuscitative Endovascular Balloon Occlusion of the Aorta for Exsanguinating Subdiaphragmatic Hemorrhage
Source: JAMA Surg. 2024 Jul 10;159(9):998–1007. doi: 10.1001/jamasurg.2024.2254 (PMC11238066; doi:10.1001/jamasurg.2024.2254)
Supplement: Supplement 1. — eFigure 1. Individual Hemodynamic responses to Z1-REBOA and P-REBOA eFigure 2. Kaplan-Meier Survival Estimates Z1 REBOA, P-REBOA and Resuscitative Thoracotomy eTable 1. Technical Outcomes – Z1 REBOA Patients eTable 2. Key Timings (minutes) [file jamasurg-e242254-s001.pdf]

Supplemental Online Content

Lendrum RA, Perkins Z, Marsden M, et al. Prehospital partial resuscitative endovascular balloon occlusion of the aorta for exsanguinating subdiaphragmatic hemorrhage. *JAMA Surg*. Published online July 10, 2024. doi:10.1001/jamasurg.2024.2254

eFigure 1. Individual Hemodynamic responses to Z1-REBOA and P-REBOA
eFigure 2. Kaplan-Meier Survival Estimates Z1 REBOA, P-REBOA and Resuscitative Thoracotomy
eTable 1. Technical Outcomes – Z1 REBOA Patients
eTable 2. Key Timings (minutes)

This supplemental material has been provided by the authors to give readers additional information about their work.

© 2024 Lendrum RA et al. *JAMA Surgery*.

eFigure 1. Individual Hemodynamic responses to Z1-REBOA and P-REBOA

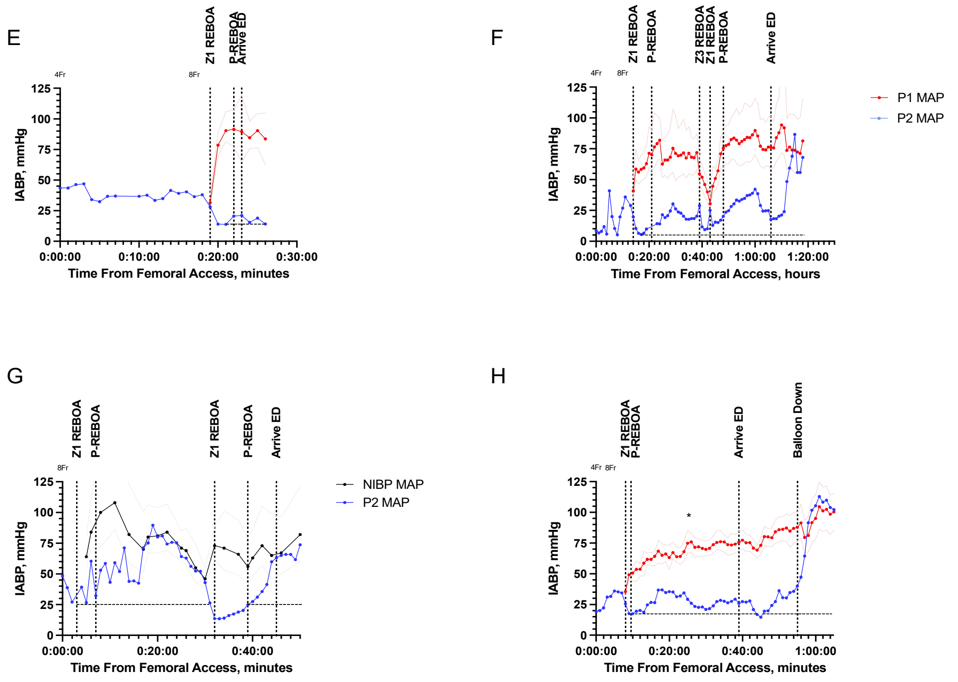

* Balloon filled with additional 0.5mls

Legend eFigure 1

P1 MAP	Proximal (aortic) mean arterial pressure measured tip REBOA catheter
P2 MAP	Distal mean arterial pressure measured side arm 8Fr access sheath CFA
NIBP MAP	Non-invasive blood pressure - mean arterial pressure
- - - - - - - - - -	Post inflation distal mean arterial pressure

E	Case 3
F	Case 6
G	Case 7
H	Case 9

© 2024 Lendrum RA et al. *JAMA Surgery.*

eFigure 2. Kaplan-Meier Survival Estimates Z1 REBOA, P-REBOA and Resuscitative Thoracotomy

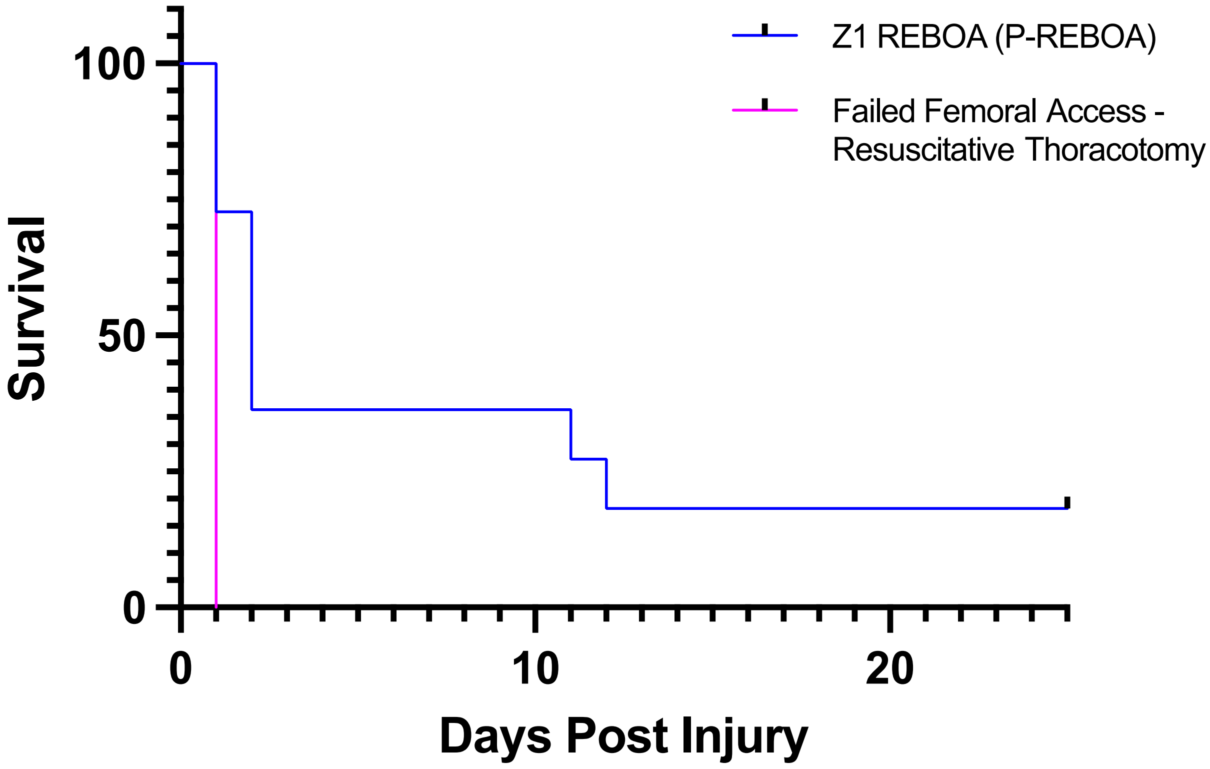

© 2024 Lendrum RA et al. *JAMA Surgery.*

eTable 1. Technical Outcomes – Z1 REBOA Patients

		Z1 REBOA				P-REBOA					Duration (min)			
Case (Sequential order)	Catheter insertion depth (cm)	Proximal IABP transduced	Balloon inflation (vol, ml)	Confirmed Z1 positioning	Repositioned (Zone)	Post inflation D-MAP mmHg (C-REBOA)	Increase D-MAP mmHg	Return distal pulsatility	Pre-hospital P-REBOA achieved	SP-REBOA	Z1 C-REBOA	Z1 P-REBOA	Z3 REBOA	Total Duration REBOA
1(1)	47	Yes	8	Yes	No	10	12	Yes	Yes	No	19	70 + 100 [J]	0	189
2 (2)	45	Yes [1]	8	Yes [a]	Yes (Z3) [D]	20	0	No	No	N/A	79	N/A	49	128
3 (3)	45	Yes	8	N/I	Yes (Z3) [D]	13	10	Yes	Yes	Yes	2	35	20	57
4 (4)	45	Yes	8	No[b]	Yes (Z3) [E]	7	14	Yes	Yes	No	10	5	65 [I]	80
5 (9)	45	Yes	8	Yes	No	15	10	Yes	Yes	No	18	102	0	120
6 (10)	45	Yes [2]	8	Yes	Yes (Z3) [E, F]	6	9	Yes	Yes	No	10 + 5 [F]	17 + 28 [F]	4	64
7 (11)	45	No [3,4]	8	Yes	No	26	30	Yes	Yes	Yes	4	88	0	92
8 (12)	45	Yes [5]	8	No [c]	No	8	0	No	No	N/A	13 [C]	N/A	0	13
9 (14)	45	Yes	5	Yes	No	18 [H]	N/A [H]	Yes [H]	Yes [H]	Yes	0	49	0	49
10 (15)	45	Yes [1]	8	Yes [a]	No	13	0	No	No	N/A	71	77 [k]	0	148
11 (16)	45	Yes	5	Yes	Yes (Z3) [G]	15	5	Yes	Yes	Yes	9	23	35	67

IABP – Intra-arterial blood pressure, N/I – Not imaged, D-MAP – Distal mean arterial pressure mmHg, P-REBOA – Partial REBOA, SP-REBOA – Spontaneous P-REBOA, N/A – Not applicable.

[1] Proximal IABP transduced post balloon inflation, monitoring setup issue (failed transducer "zero" therefore no numerical value recorded), [2] Prolonged blockage access sheath and therefore lack of distal pressure value, [3] multiple set-up issues transducer on REBOA catheter lumen proximal to balloon (balloon transducer 3-way tap open to air/atmospheric pressure, failure pressurization REBOA catheter flush line, ultimately failure of arterial transducer requiring replacement on hospital arrival, NIBP values subsitituted) [4] Inadvertent balloon 3-way tap displacement resulting in balloon deflation and hypotension, [5] Proximal IABP transduced post REBOA (transducer cable disconnection).
[a] Surgical palpation, [b] Repositioned – Z3 pre-hospital (peri-renal (Z2) on CT, [c] Pronounced Life Extinct on scene, device in situ, [D] Operating theatre, [E] Pre-hospital, [F] Repositioned Z1 due to hypotension, [G] Emergency Department, [H] Straight to P-REBOA, no period C-REBOA, distal pulsatility never lost, [I] 13 minutes Z3 C-REBOA + 52 minutes Z3 P-REBOA, [J] Second period Z1 P-REBOA during repeat DCS Laparotomy (approximate timings), [k] in-hospital P-REBOA, commenced in the ED (approximate timings) following titrated balloon deflation.

© 2024 Lendrum RA et al. *JAMA Surgery.*

eTable 2. Key Timings (minutes)

Case (Sequential order)	Emergency call – REBOA	LAA arrival – REBOA	REBOA – P-REBOA	LAA Scene Time	Emergency call – Hospital Arrival
**REBOA**					
1 (1)	54	10	6	32	89
2(2)	67	49	N/A	67	108
3(3)	74	62	2	49	72
4(4)	56	31	10	39	73
5(9)	53	34	18	41	86
6(10)	67	38	10	77	115
7(11)	54	28	4	56	93
8(12)	54	30	N/A	59	N/A
9(14)	57	40	0	55	82
10(15)	74	43	N/A	56	115
11(16)	67	54	9	60	89
**Failed femoral arterial access**					
12(5)				47	84
13(6)				48	85
14(13)				32	94
**EFA only**					
15(7)				71	111
16 (8)				50	96
**Median (IQR)**	57 (54-67	38 (30-49)	8 (3-18)	**Cohort** 53 (43-60)<br>**REBOA** 55 (47-67)	**Cohort** 89 (84-108)<br>**REBOA** 89 (82-108)

© 2024 Lendrum RA et al. *JAMA Surgery.*
