# Supplementary material for: Prehospital Partial Resuscitative Endovascular Balloon Occlusion of the Aorta for Exsanguinating Subdiaphragmatic Hemorrhage
Source: JAMA Surg. 2024 Jul 10;159(9):998–1007. doi: 10.1001/jamasurg.2024.2254 (PMC11238066; doi:10.1001/jamasurg.2024.2254)
Supplement: Supplement 2. — Data sharing statement [file jamasurg-e242254-s002.pdf]

Data Sharing Statement

Lendrum. Prehospital Partial Resuscitative Endovascular Balloon Occlusion of the Aorta for Exsanguinating Subdiaphragmatic Hemorrhage. *JAMA Surg*. Published July 10, 2024. doi:10.1001/jamasurg.2024.2254

Data
Data available: Yes
Data types: Deidentified participant data
How to access data: Data will be made available for sharing on request. How to access data: Requests for access should be addressed to the corresponding author and will be considered by the study team. robert.lendrum@nhs.net
When available: With publication

Supporting Documents
Document types: Other (please specify)
Additional Information: Study Protocol
How to access documents: Requests for the protocol should be addressed to the corresponding author. robert.lendrum@nhs.net
When available: With publication

Additional Information
Who can access the data: Researchers whose proposed use of the data has been approved.
Types of analyses: These will be considered by the study team.
Mechanisms of data availability: After approval of a proposal.
